# An integrative genomic analysis of transcriptional profiles identifies characteristic genes and patterns in HIV-infected long-term non-progressors and elite controllers

**DOI:** 10.1186/s12967-019-1777-7

**Published:** 2019-01-21

**Authors:** Jiwei Ding, Ling Ma, Jianyuan Zhao, Yongli Xie, Jinming Zhou, Xiaoyu Li, Shan Cen

**Affiliations:** 1 Institute of Medicinal Biotechnology, Chinese Academy of Medical Sciences, Beijing, 100050 China; 2 Key Laboratory of the Ministry of Education for Advanced Catalysis Materials, Department of Chemistry, Zhejiang Normal University, Jinhua, Zhejiang 321004 China

**Keywords:** Elite controller, Long-term nonprogressor, Viremic nonprogressor, Meta-analysis, GSEA, WGCNA

## Abstract

**Background:**

Despite that most HIV-infected individuals experience progressive CD4+ T cell loss and develop AIDS, a minority of HIV-infected individuals remain asymptomatic and maintain high level CD4+ T cell counts several years after seroconversion. Efforts have been made to understand the determinants of the nonprogressive status, exemplified by the clinical course of elite controllers (ECs) who maintain an undetectable viremia and viremic nonprogressors (VNPs) who have a normal CD4+ count in spite of circulating viral load. However, the intrinsic mechanism underlying nonprogression remained elusive. In this study, we performed an integrative analysis of transcriptional profiles to pinpoint the underlying mechanism for a naturally occurring viral control.

**Methods:**

Three microarray datasets, reporting mRNA expression of the LTNPs or ECs in HIV-infected patients, were retrieved from Gene Expression Ominbus (GEO) or Arrayexpress databases. These datasets, profiled on the same type of microarray chip, were selected and merged by a bioinformatic approach to build a meta-analysis derived transcriptome (MADNT). In addition, we investigated the different transcriptional pathways and potential biomarkers in CD4+ and CD8+ cells in ECs and whole blood in VNPs compared to HIV progressors. The combined transcriptome and each subgroup was subject to gene set enrichment analysis and weighted co-expression network analysis to search potential transcription patterns related to the non-progressive status.

**Results:**

30 up-regulated genes and 83 down-regulated genes were identified in lymphocytes from integrative meta-analysis of expression data. The interferon response and innate immune activation was reduced in both CD4+ and CD8+ T cells from ECs. Several characteristic genes including CMPK1, CBX7, EIF3L, EIF4A and ZNF395 were indicated to be highly correlated with viremic control. Besides that, we indicated that the reduction of ribosome components and blockade of translation facilitated AIDS disease progression. Most interestingly, among VNPs who have a relatively high viral load, we detected a two gene-interaction networks which showed a strong correlation to immune control even with a rigorous statistical threshold (p value = 2−e4 and p value = 0.004, respectively) by WGCNA.

**Conclusions:**

We have identified differentially expressed genes and transcriptional patterns in ECs and VNPs compared to normal chronic HIV-infected individuals. Our study provides new insights into the pathogenesis of HIV and AIDS and clues for the therapeutic strategies for anti-retroviral administration.

**Electronic supplementary material:**

The online version of this article (10.1186/s12967-019-1777-7) contains supplementary material, which is available to authorized users.

## Background

Despite the great progress in combination anti-retroviral therapy (cART), AIDS is still a non-curative disease that causes a high death rate every year. It is interesting that there are a small proportion of HIV-infected individuals who maintain a stable CD4+ T cell count within the normal reference range over several years from the diagnosis of HIV infection in the absence of ART and clinical symptoms. These patients with spontaneous and sustained control of HIV disease progression were identified as long term nonprogressors (LTNPs) [1, 2]. Similarly, there are approximately 1% of HIV-infected patients who maintain undetectable viral load for a prolonged period (generally less than 50 copies/ml), who are identified as elite controllers. These two groups of patients classified by the immunologic parameters or viral parameters represent useful models of natural protection against disease progression and may have important implications for prophylactic and therapeutic strategies [3]. Thus far it is still in debate whether viral factor, host or environmental factor contributes to the LTNP status [2, 4]. Association of augmented control with attenuated viruses due to deletions or mutations of regulatory proteins or other special viral polymorphism have been reported by several researches [5–8]. For instance, a F72L mutation in HIV-1 Vpr in a LTNP was reported to confer a significant reduction in Vpr nuclear import and virion incorporation implying a link between efficient Vpr nuclear import and HIV disease progression [9]. Moreover, viruses isolated from some LTNPs were found to have gross deletions in nef genes, suggesting the importance for nef gene in AIDS disease progression [10, 11]. However, studies of nef genes in elite controller yield contradicting results [12, 13]. A phylogenetic analysis revealed that nef sequences from patients with different rates of progression did not form distinct cluster between LTNP and progressors, suggesting the degree of variation in nef is unlikely to be indicative of disease progression [14, 15].

Cellular immune responses have also been reported to contribute to viral control [16, 17]. For instance, a tight association was observed between Gag specific CD8+ T cells and viral control [17–20]. Some elite suppression was linked with higher level of cytolytic granules within HIV-specific CD8+ T cells [21]. Additionally, CD8+ T cells isolated from ECs exhibited more polyfunctional capability in response to HIV specific antigens [22–25]. On the other hand, CD4+ T cells from ECs retained an ability to proliferate and produce interleukin 2 (IL-2) in response to HIV compared with normal patients under ART [26]. In addition to CD4+ and CD8+ T lymphocytes, a correlation of Interleukin 17 secreting T (Th17) cell level and HIV disease progression was observed in LTNPs compared to TPs, supporting a role of this cell subset in HIV pathogenesis [27]. Besides, humoral immune response also plays a role in the context of natural viral control. Some researchers have reported that ECs maintained HIV-1 specific memory B cell response which contributed to neutralizing responses in contrast to treated patients [28].

Moreover, various approaches have been undertaken to uncover the host genetic factors or specific genes involved in virologic control of HIV infection in ECs [29, 30]. A study showed a role of p21 in ECs via indirectly blocking reverse transcription by inhibiting CDK2-dependent phosphorylation [31]. The HLA class I allele HLA-B*57 [32, 33] and HLA-B*27 alleles [34, 35] were overrepresented among elite controllers and viremic controllers (VCs) compared with normal progressors, which underscored the important role of CD8+ T cells in naturally viral control. [25]. Most notably, HLA class I molecules might affect HIV-1 immune control by interacting with their receptors on innate immune cells, such as the killer cell immunoglobulin-like receptors (KIR) on natural killer (NK) cells. Two studies reported that activating KIR3DS1 allele in combination with Bw4-80I and KIR3DL1*004 in the presence of HLA-Bw4 showed a pronounced protection against AIDS progression [36, 37]. Genome-wide association studies have been carried out to decipher the association between naturally occurring single nucleotide polymorphism (SNPs) and viremic control in ECs and LTNPs [38, 39]. An international HIV controllers study identified over 300 SNPs on chromosome 6 involved in viral control [39]. However, only approx. 20% of the protective effects can be attributable to the SNPs discovered, indicating other unknown mechanisms were accountable for the observed control. Further studies are necessary to pinpoint more novel pathways and intrinsic host factors responsible for virological control.

In most studies investigating the host intrinsic factors controlling disease progression, only a few ECs or LTNPs were involved and the conclusions are often controversial. To overcome this limitation of individual studies, a large-scale transcriptional study was necessary to reduce random error and increase statistical power. In this study, we combined relevant microarray data to increase statistical power to uncover the biological differences between LTNP or ECs and chronic progressors. To decrease the heterogeneity and increase the consistency between different datasets, we only select microarray data from Human Genome U133A or Human Genome U133 plus 2 Array (Affemetrix Company). Furthermore, a new illumina data series GSE87620 was used as a validating set. To be most informative, we conducted our analysis in two steps. First, we combined the three datasets into meta-analysis derived transcriptome, which provided a comprehensive comparison of nonprogressors (NP) with progressors (PP). Second, we split these study subjects into three subgroups, namely CD4+ T cell samples from ECs, CD8+ T cell samples from ECs and whole blood samples from VNPs, thereby providing an elaborate comparison between aviremic controllers or viremic controllers and progressors. Differential expression analysis, gene set enrichment analysis and WGCNA approach were carried out in each step. Our study have revealed some characteristic biomarkers and transcriptional patterns and highlight several key genes in nonpathogenic individuals, Most strikingly, we identified key transcriptional modules in VNPs which have never been reported before. These findings may better the understanding of HIV-1 viremic and immune control and AIDS progression.

## Methods

### Selection of studies and datasets

Expression profiling studies including LTNPs or ECs were identified through Gene Expression Omnibus (GEO, http://www.ncbi.nlm.nih.gov/geo) using search term (“long term no progressor” OR “elite controller”). To ensure the relevant studies were not missed, search in Arrayexpress (http://www.ebi.ac.uk/arrayexpress) was also performed. Nine microarray gene expression datasets, reporting expression data of LTNPs or ECs and normal patients were retrieved from public repositories. Three datasets (GSE24081, GSE6740 and GSE57730) profiling on the same version of microarray chips [Human Genome U133 Plus 2.0 Array (HG-U133_Plus_2) or Human Genome U133A ver2.0 (U133A)], met the inclusion criteria and were included in the integrated analysis to build a MADNT set. The characteristics of these datasets were listed in Table 1. Detailed clinical parameters of each patient involved were listed in Additional file 1: Table S1.

**Table 1:** General information of each dataset

First author	GEO number	Platform	Sample source	Sample size (PP/NP)
Martin Hyrcza	GSE6740	GPL96; Affymetrix Human Genome U133A Array	CD4+ T cell CD8+ T cell	PP = 10; NP = 10 *3 NPs are ECs and 2 with extremely low viral load
W. Nicholas Haining	GSE24081	GPL3921; Affymetrix HT Human Genome U133A Array	CD8+ T cell	PP = 18;NP = 24 *All NPs are ECs
Gregory K Tharp	GSE57730	GPL570; Affymetrix Human Genome U133 Plus 2.0 Array	Whole blood	PP = 7; NP = 5 *All NPs are VNPs

*PP* progressor, *NP* nonprogressor, *EC* elite controllers, *VNP* viremic nonprogressor

### Data processing

Microarray meta-analysis were carried out according to the guidelines described in [40]. Each datasets were log2 transformated and normalized by Agilent GeneSpring software (Version 11.5, Agilent, USA). Then, gene matching was done for all probes. When multiple probes matched the same gene symbol, the probe presented the greatest inner-quartile range (IQR) was selected to represent the target gene symbol. After matching all the probes to a common gene symbol, “MetaDE” R package [41] was exploited to merge the common gene symbols across multiple studies by p value combination using Fisher methods. Differentially expressed genes were selected with adjusted *p* value < 0.05, based on false discovery rate (FDR) by the Benjamini–Hochberg procedure and moderated t test.

### Enrichment analysis

Enrichment analysis for KEGG pathway and Gene Ontology terms were carried out by David online tool (https://david.ncifcrf.gov). Gene set enrichment analysis (GSEA) [42] was carried out using GSEA version 3.0, downloaded from the Broad Institute (http://www.broadinstitute.org/gsea/downloads.jsp). Expression data sets and phenotype labels were created according to GSEA specifications. Gene set permutations were set to be done 1000 times for each analysis using the weighted enrichment statistic and signal to noise metric. Gene sets with FDR lower than 0.05 were considered significant.

### WGNCA

Weighted gene coexpression network analysis (WGCNA) is a gene coexpression network-based approach [43, 44]. A gene co-expression network is defined as undirected, weighted gene network, in which the nodes represent expression profiles while edges represent pairwise correlation between gene expressions. Briefly, correlation coefficient Smn between characteristic gene m and gene n is calculated by their expression values between different samples using the formulation: Smn = |cor(mn). The correlation matrix was then transformed into an undirected network by raising the absolute value of each entry to a power β using 6 as correlation coefficient threshold. Genes were clustered into different modules using dynamic tree cutting method.

Protein–protein interactions (PPI) networks in each module were visualized by Cytoscape 3.6.0. The Network Analyzer examined the network for topological parameters, including degree, connectivity, betweenness and closeness.

### HIV infection assay and western blotting

To analyze the antiviral activity of several up-regulated genes, HIV infection assay was performed as previously described [45, 46]. Briefly, 200 ng pNL4-3-R-E-Luc vector and 150 ng pCMV-VSV-G packaging vector were transfected into the 293T cells together with 200 ng empty pCMV6-Entry vector, control gene (SLFN11, GADD45G) or pCMV6-Entry-CMPK1, ZNF395, METTL9, GADD45A, PKKAR2B, OAT, CHPT1, SPOCK2 and CBX7 in 12-well plate using Lipofectamine 2000 (Thermo Scientific) according to the manufacturer’s instructions. The old culture medium was replaced with fresh culture medium at 6 h post-transfection. Subsequently, the supernatants were collected at 48 h post-transfection and cleared by filtration with the 0.45 μm filter. Cell lysis were harvested for SDS-PAGE and western blotting with α-myc, p24 (Gag) and β-actin antibody, respectively. The amount of (infectious) virus particles in the supernatants was determined via one-cycle infection assay. For infection assays, 200 μl of 1 × 10^5^ SupT1 or Jurkat cells per well in 96 well plate were infected with supernatants collected from 293T cells. At 48 h post-infection, the SupT1 cells were lysed and luciferase activity was determined using a firefly Luciferase Assay System (Promega).

293T cells were maintained at 37 °C in high glucose Dulbecco’s modified Eagle medium (DMEM) supplemented with 10% fatal bovine serum (Gibco). SupT1 and Jurkat cells were maintained at RPMI 1640 Medium with 10% fetal bovine serum.

## Results

### Description of datasets

We searched two public repositories for mRNA transcriptional profile related to viremic control using the search term” “elite controller” [OR] “long-term nonprogressors”. The inclusion criteria were set as (1) the microarray chips used in the study was from Human Genome U133A or Human Genome U133 plus 2 Array (Affemetrix Company); (2) sample source was CD4+, CD8+ T cells or whole blood. Three studies met the inclusion-criteria were selected for the meta-analysis study. Details about these datasets were outlined as in Table 1. The three series included 35 PPs, 34 ECs (except for two patients with extremely low viremia) and 5 VNPs. The following information was extracted from each data series including GEO number, author name, sample source, platform and the number of progressors and controllers.

### Microarray meta-analysis

**Fig. 1:**
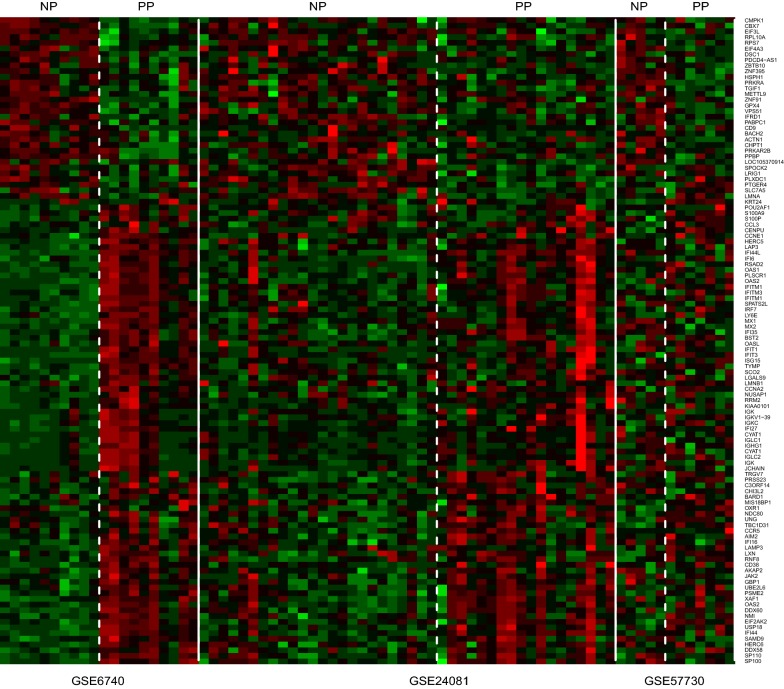
The heatmap plots of DEGs between nonprogressors and progressors visualized using MetaDE package. Each row represents a gene and each line represents a sample; NP and PP denote nonprogressor and progressor, respectively. The white sold lines separate two adjacent studies. The white dotted lines separate the nonprogressors and progressors. The top 113 genes (FDR < 0.05) are shown. Red represents higher expression and green represents lower expression

To compile expression data for meta-analysis, Cel raw data was firstly preprocessed by GeneSpring 11.5 software (Agilent Technologies, Santa Clara, CA). The background subtraction, normalization, and log base 2 transformation of gene signals were carried out using Robust Multi-Array Analysis (RMA) summarization algorithm. Quality of each dataset was assessed in Genespring and expression data was retrieved from GeneSpring. MetaDE packages were exploited to combine the three datasets and differentially expressed genes (DEG) in NPs compared to normal chronic progressors. DEG p value in each study was calculated by moderated-t statistical analysis. Fisher combined probability test was chosen for meta-analysis statistical method. For statistics, Benjamini and Hochberg method was used for multiple testing correction. Genes with FDR less than 0.05 was accepted as DEGs. We found 30 upregulated DEG and 83 down-regulated DEGs in NPs compared with PPs (Fig. 1). Up-regulated genes included CMPK1, CD9, METTL9, EIF4A3, ZNF395 and so on, while down-regulated genes were overrepresented by CD38, LAG3, some Interferon-stimulated genes (ISGs) and inflammatory genes. Upregulated and down-regulated DEGs in the comparison of NPs and PPs were shown in Additional file 2: Table S2.

**Fig. 2:**
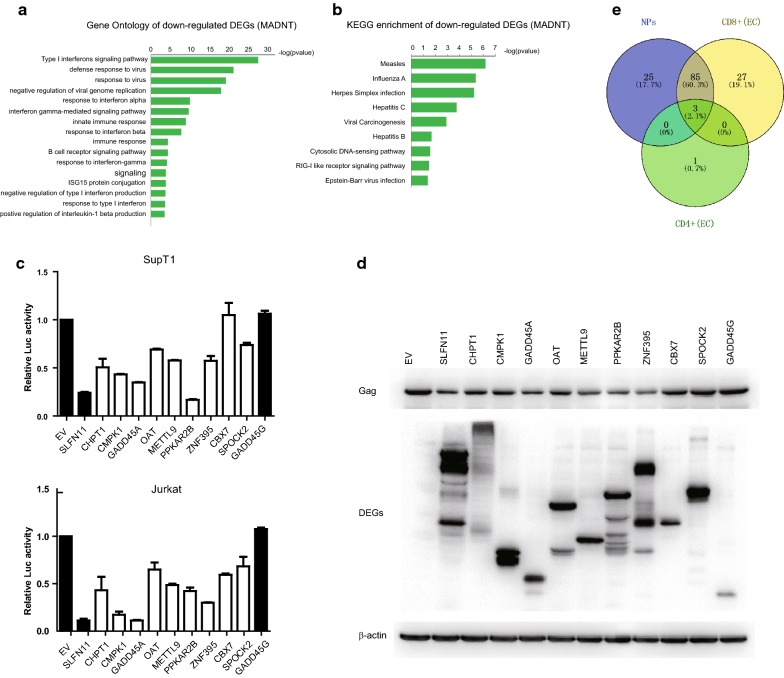
Functional annotation of down-regulated genes and anti-HIV activity of up-regulated genes. **a** Gene Ontology of down-regulated DEGs in MADNT. **b** KEGG pathway enrichment of down-regulated DEGs in MADNT. **c** Validation of anti-HIV activity of night selected up-regulated DEGs. HEK293T cells were transfected with pNL4-3luc.R-E-, VSV-G with myc tagged-CMPK1, METTL9, GADD45A, CHPT1, OAT, PPKAR2B, ZNF395, SPOCK2, CBX7 or empty vector or control gene (GAD45G and SLFN11), 48 h post-transfection, virions in the supernatant were filtered and used for infecting SupT1 and Jurkat cells. 48 h post-infection, luciferase activity in SupT1 cells (upper panel) and Jurkat cells (lower panel) were measured. Black columns represent controls and white columns represent DEGs. EV denotes empty vector. **d** Gag expression influenced by these up-regulated DEGs. The expression of HIV Gag, myc-tagged DEGs and β-actin were determined by Western blotting. **e** Venn. Diagram of DEGs from CD8+ and CD4+ T cells of ECs and all NPs

Gene Ontology (GO) and Kyoto Encyclopedia of Genes and Genomes (KEGG) analysis were performed for the functional evaluation of both up-regulated and down-regulated DEGs using DAVID (Database for Annotation, Visualization and Integrated Discovery) online tool. In regard to GO analysis, “type 1 interferon signaling pathway”, “defense response to virus”, “interferon-gamma mediated-signaling pathway”, “innate immune response” and “B cell signaling pathway” were enriched in down-regulated genes (Fig. 2a). In the pathway analysis, several anti-viral response pathways was enriched, including “Measles”, “Influenza A”, “Hepatitis B” and “Hepatitis C” (Fig. 2b). The analysis failed to enrich GO terms or KEGG pathways with FDR less than 0.05 in up-regulated genes. Unlike down-regulated genes, the biological meanings with up-regulated genes were sporadic and their relevance to AIDS disease progression was not immediately clear. Furthermore, night genes (CMPK1, METTL9, CHPT1, OAT, GADD45A, SPOCK2, CBX7, ZNF395 and PPKAR2B) were selected to validate the anti-HIV activity. 293T cells were transfected with NL4-3lucR-E- and VSV-G along with these genes, empty vector or control genes (GADD45G, SLFN11). 48 h post-transfection, the supernatant was harvested and used for infecting SupT1 and Jurkat cells. After another 48 h, luciferase activity in SupT1 or Jurkat cells was measured to assess the anti-HIV activity. CMPK1, GADD45A, PPKAR2B and CHPT1 suppressed HIV-1 production by more than twofold. METTL9, ZNF395 SPOCK2 and OAT exerted a moderate inhibitory effect on HIV-1 production. Notably, GADD45A inhibited HIV-1 production by more than eightfold in Jurkat cells (Fig. 2c). GADD45G, the expression of which varied little between NPs and PPs was used here as a negative control gene and showed no effect on HIV replication. SLFN11 which was reported to inhibit HIV-1 production in the previous studies was used here as a positive control [47]. To further investigate whether these up-regulated DEGs influences the expression of HIV gene, Gag expression in 293T cells was assessed by Western blotting. CHPT1, CMPK1, GADD45A, OAT, METTL9, PPKAR2B and ZNF395 significantly decreased the expression of HIV-1 Gag (Fig. 2d). The functional assay confirmed the reliability of our analysis results.

A pilot study reported that serum from LTNPs and ECs have distinct neutralizing capabilities and antibody-dependent cellular cytotoxicity (ADCC) activity [48]. To uncover the divergent transcription pattern between aviremic controllers and VNPs from different cell types, study subjects were grouped into three subsets aforementioned. Data of CD8+ T cells from two datasets were merged into a combined transcriptome by MetaDE package. 115 DEGs were identified in CD8+ T cells (Additional file 3: Table S3), which highly overlap with those identified in MADNT. Only 4 DEGs (OAS3, IFI44, IFI44L and EIF2AK2) were identified in CD4+ T cells of ECs (Fig. 2e), and no DEGs met the criteria (FDR < 0.05) in VNPs.

Taken together, statistical combination of the relevant datasets has created a meta-analysis derived nonprogressor transcriptome (MADNT) and different subgroups, which can be subject to downstream GSEA and WGCNA analysis.

### Gene set enrichment analysis

**Fig. 3:**
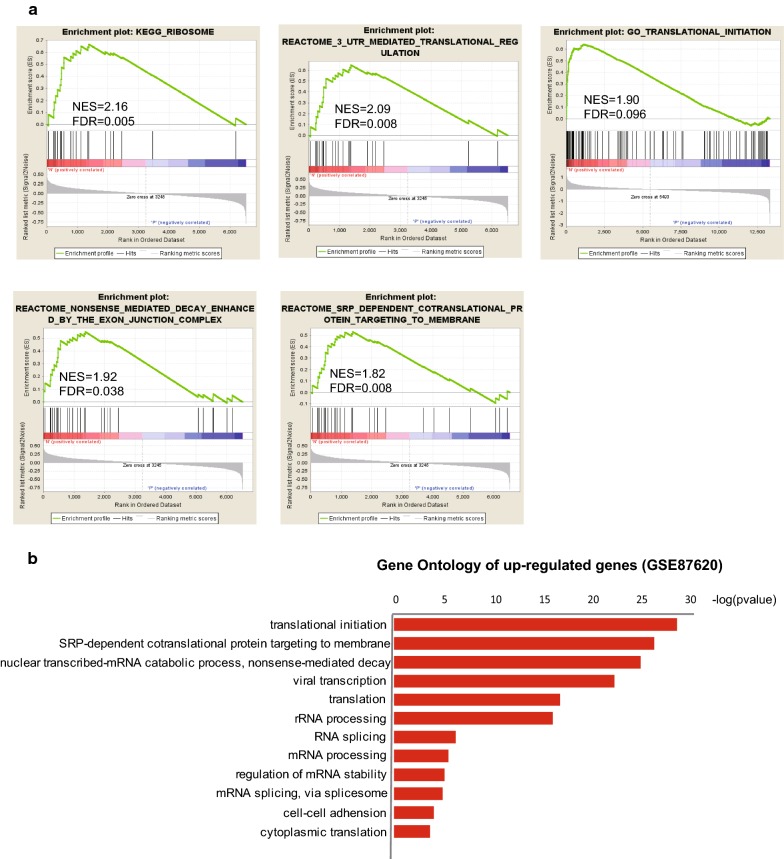
GSEA analysis of MADNT expression data. **a** Prominent transcriptional patterns in NPs. The diagram showed GSEA plots for 5 pathways recapitulating the main difference between NPs and PPs (NPs were shown at left-hand side in GSEA plot, PPs right-hand side). The vertical axis in the upper graph indicates Enrichment Score (ES) for genes in each gene set. The barcode plot indicates the position of genes in each gene set. Red and blue colors represent positive and negative Pearson correlation with HIV progression. **b** Functional annotation of up regulated genes with FDR less than 0.05 in the validating set (GSE87620)

Given the limited power to detect the transcriptional pattern in up-regulated genes in LTNPs and ECs, GSEA was carried out to investigate the intrinsic common features associated with virological or immunologic control. Rather than setting a cutoff value for single DE genes, GSEA evaluates the whole dataset at biological pathway level by performing unbiased global search for predefined gene sets. MADNT and different subgroups mentioned above were interrogated for pathway enrichment using canonical pathway from MSigDb 2 GO-BP collection and also C2 (KEGG and Reactome) collection. Fourteen pathways were enriched with FDR less than 0.05 in NPs which included “KEGG_RIBOSOME”, “3′UTR mediated translational regulation”, “Nuclear transcribed mRNA catabolic process nonsense-mediated decay“, ”SRP-dependent cotranslational protein targeting to membrane” and “translational initiation” (Fig. 3a). Besides, we noticed that KEGG pathway “Graft verse host disease” was also enriched at moderately significant level (FDR around 0.19 and nominal p < 0.03), which included HLA-DQB1, HLA-DQA1, HLA-DOB, HLA-DMA and HLA-DOA. More than 30 pathways were enriched in progressors, which included “responses to type I interferon”, “mitotic cell cycle”, “defense response to virus”, “innate immune response” and “regulation of interleukin_1 beta production”. Furthermore, to validate the reliability of these datasets from Affemetrix microarray, we selected another dataset (GSE87620) published recently, using illumina Human HT-12 V4 microarrays. Using GSE87620 as a validating set, we selected the up-regulated genes with FDR < 0.05 using online tool GEO2R and performed Gene Ontology annotation (Fig. 3b). The enriched pathways were highly consistent with the results from MADNT. “translational initiation”, “SRP-dependent cotranslational protein targeting to membrane” and “nuclear-transcribed-mRNA catabolic process, nonsense-mRNA decay” was enriched in NPs.

In the aspect of stratification subgroup, 6 pathways were enriched with FDR less than 0.25 in CD4+ T cells of ECs which included “multi organism metabolic process”, “translational initiation”, “Nuclear transcribed mRNA catabolic process nonsense-mediated decay”, “protein targeting to membrane”; Similarly, 32 pathways were enriched with FDR less than 0.01 in CD8+ T cells of ECs, most of which were associated with pathways related to translation and protein-targeting to membrane (Additional file 4: Table S4).

### Weighted gene co-expression network analysis

WGCNA provides insights to disease pathogenesis by studying co-expression genes between clinical samples based on gene expression correlation coefficients. The reliability of this algorithm depends on a large sample size, which we are able to apply the MADNT and two subgroups (CD8+ T cells (EC) and VNP) expression set into WGCNA analysis.

**Fig. 4:**
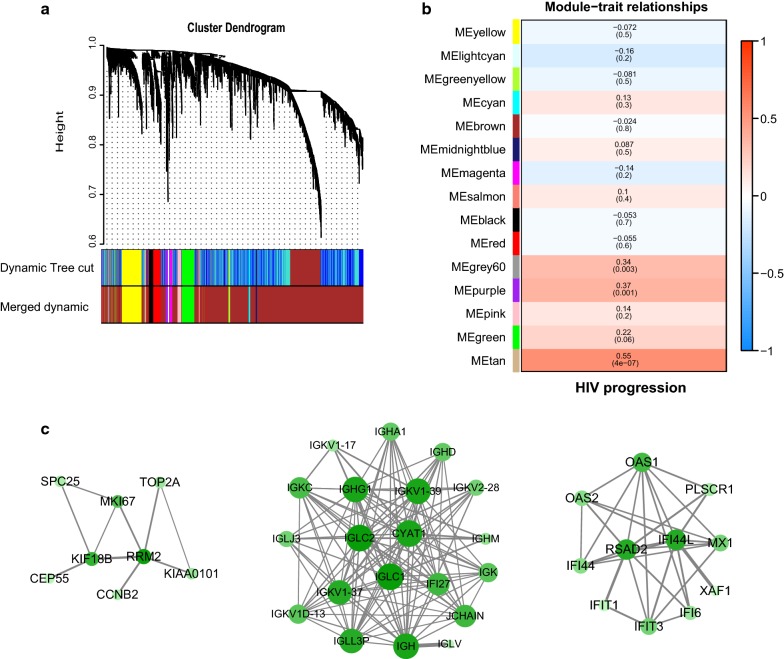
WGCNA analysis of MADNT expression data. **a** Gene co-expression modules. Genes that can not be clustered into one of these modules are assigned to the grey module. Every gene represents a line in the hierarchical cluster. Distance between two genes is shown as height in the y axis. Different colors represent different modules. **b** Module-trait relationship. The top value in each square shows the correlation between the module eigengene and the outcome (disease progression). The brightness of the color means the tightness of correlation. The bottom value is p value of each correlation. **c** Visualization of the three modules (left: grey60 module; middle: purple module; right: tan module) highly correlated with disease progression with p value less than 0.05 by Cytoscape. Green nodes denote down-regulated genes in NPs. The color intensity of each node represents the centrality of gene in the network. The width of each edge represents betweenness

Using WGCNA, 15 distinct modules was found (Fig. 4a). The expression data from different genes within each calculated module was used to determine the module epigengenes (ME, the first component of the respectively module), which was correlated to the clinical parameter (AIDS progression). Several modules showed significant positive correlation with lose of viremic control, including the tan modules (p = 4e−07, correlation coefficient = 0.55) and purple modules (p = 0.001, correlation coefficient = 0.37) and grey60 module (p = 0.003, correlation coefficient = 0.34) (Fig. 4b). However, no modules were obviously correlated to viremic control. For the three modules related to disease progression, 39 characteristic genes were subject to functional annotation and pathway enrichment. They were totally enriched in 19 GO terms. The genes were largely related to type I interferon signaling pathway (IFIT1, IFIT3, IFI6, OAS1, OAS2, MX1), complement activation (IGHA1, IGHD, IGHG1, IGHM, IGKV1-17, IGKV1-37, IGKV1-39) and positive regulation of B cell activation (IGHA, IGHD, IGKC, IGLC1). Additionally, PPI networks of the three modules were visualized by Cytoscape 3.6 (Fig. 4c). RRM2, IGLC2 and RSAD2 were the hub genes of grey60, purple and tan modules, respectively.

**Fig. 5:**
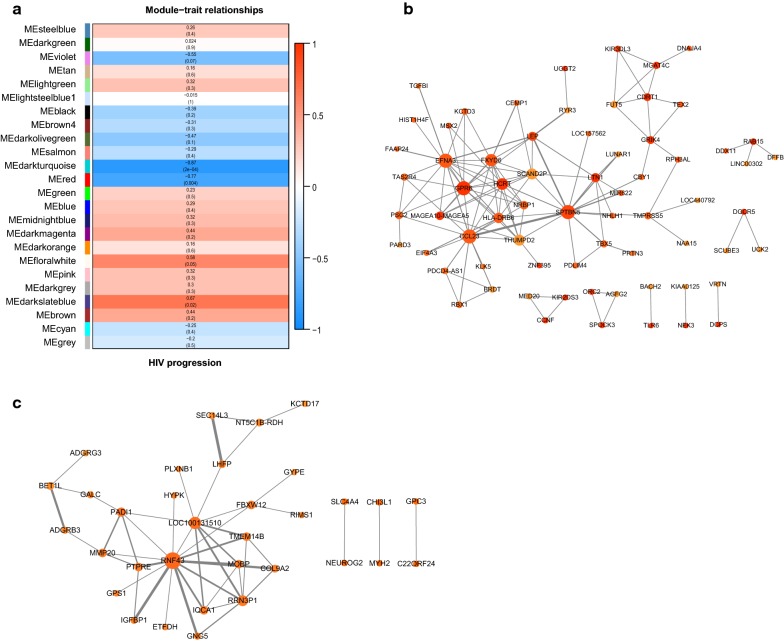
WGCNA analysis of VNP transcriptome. **a** Module-trait relationship. The top value in each square represent the correlation between the module eigengene and the outcome (disease progression). The brightness of the color means the tightness of correlation. The bottom value is p value of each correlation. Visualization of darkturquiose module (**b**) and red module (**c**) which was highly correlated with disease progression with p value less than 0.05 by Cytoscape. Orange nodes denote up-regulated genes in VNPs. The color intensity of each node represents the centrality of gene in the network. The width of each edge represents betweenness

As to the stratification subgroup, WGCNA approach was applied to CD8+ T cells of ECs and VNPs, respectively. We identified 24 distinct coexpression modules in CD8+ T cells containing 28 to 1174 genes per module. Salmon module showed a strong positive correlation with AIDS progression including CD38 and ISGs which is consistent with DEG and GSEA analysis. Magenta and purple module (Additional file 5: Table S5) showed a positive correlation with viral control in ECs including gene involved in regulation of cellular response to stress (DNAJA1, HSPA5, PIK3R1 and PMAIP1), leukocyte migration (SCL7A5, YES1, B4GALT1, NRAS, PDE4B, PIK3R1 and PODXL) and response to cytokines (REL, SKIL, MAPKAPK2 and NFKB2). Interestingly, two modules were significantly associated with immune control in VNPs (darkturquoise, ME correlation = 0.87, p-value = 2e−4; red, ME correlation = 0.77, p-value = 0.004) (Fig. 5a). ZNF395, PDCD4-AS1 and EIF4A3 that are among the 30 up-regulated genes were involved in the darkturquoise module, Notably, KIR2DS3 and KIR3DL3 which were reported to affect immune control of HIV by interacting with HLA-B molecules in a specific manner also appeared in the darkturquoise module. We failed to enrich GO or KEGG terms of these modules, probably indicating unknown important pathways or interactions between genes in these two modules accounting for the immune control in VNPs. PPI networks of the two modules were visualized by Cytoscape 3.6 (Fig. 5b, c).

## Discussion

Increasing transcriptional profiles allowed us to investigate the significant genes and functional pathways relevant to viral control in ECs and VNPs in a large scale level. In this study, we adopted an integrative approach to uncover the transcriptional patterns in LTNPs and ECs and identified several key genes and functional pathways which are significantly connected to viral control and disease progression.

We found several common genes up-regulated in all ECs and VNPs, some of which have never been reported before, including ZBTB10, ZNF395, GPX4, CHPT1, METTL9, SPOCK2, EIF4A3, GADD45A, SLC75A, TGIF1, CMPK1, LMNA, PLXDC1 and BACH2. GADD45A is a stress-induced protein and participate in various cellular pathways. A recent study reported that overexpression of GADD45 proteins reduces HIV-1 production through suppressing transcription from the HIV-1 LTR promoter [49]. We have experimentally examined several up-regulated genes for their anti-HIV activity in cell lines, most of which were demonstrated to suppress the replication of VSVG-pseudotyped HIV-luc viruses. The biological relevance of these up-regulated genes in the context of HIV pathogenesis needs further investigation.

On the other hand, an arsenal of ISGs were down-regulated in NPs compared to chronic progressors, which further debates the role of interferon in chronic HIV infection. Several researches by other groups observed similar phenomenon. Margalida Rotger showed several ISGs (IF44, MX1, EIF2AK2, IFI6, LY6E, TRIM22) were up-regulated in RPs who got a progressive immunosuppression soon after seroconversion while a lower expression of ISGs was observed in CD8+ T cells in viremic nonprogressor (VNP) profile [50]. Sankaran et al. reported a significant increase in gene expression regulating immune activation and inflammatory response in intestinal mucosa in HIV-infected patients with high viral load compared to LTNPs [51]. In accordance with this, CD38 and LAG3 which were found to be elevated in RPs have also been identified in our analysis. Consistent with this knowledge, a research reported a significant increase of ISG expression with increased viral load, including genes of intrinsic antiretroviral defense [52]. Currently, the contribution of IFN-Is to the control of viral infection and to the immunopathogenesis of AIDS is still under debate [53, 54]. Supportively, Liang [55] implied that type I interferon contributed to aberrant immune activation, T cell depletion and dysfunction during chronic HIV-1 infection. They found that persistent HIV-1 infection in humanized-mice led to induction of IFNs and ISGs including MX2, IFITM3, Trim22, ISG15, OAS1, and IFN regulatory factor 7 (IRF7) both in peripheral blood mononuclear cells (PBMCs) and in the spleen. They also observed the enhanced expression of CD38 and HLA-DR, similar to our data. Persistent immune activation plays a central role in CD4+ T cell depletion and progression to AIDS and may be considered to be a predictor of disease progression in ART-naïve patients. Taken together, our analysis supports the concept that IFNs and ISGs are a double-edged sword during chronic infection and highlighted the caution of IFNs usage in HIV-1 chronically infected patients.

Given the limited power of classical function annotation, GSEA and WGCNA algorithm was performed on our combined transcriptional profile individually. Pathways including “translational initiation”, “3′UTR mediated translational regulation” and “nuclear transcribed-mRNA nonsense-mediated decay” were shown to be positively correlated with viremic control. In contrast, pathways related to interferon and immune response, complement activation and cell cycle seemed to be highly correlated with AIDS disease progression. Notably, ribosomal substitutes including RPS20, RPS28, RPS15A, RPS25, RPS6, RPS21, RPS3, RPL36, RPL9, RPL31, RPL23, RPL27, RPL30, RPL29, RPL35 and translational initiation factors including EIF2S3, EIF1, EIF2C2, EIF4G2, EIF4A3 were elevated in NPs compared with chronic progressors by GSEA analysis. This indicates that defects in ribosome components and blockade of translational initiation probably play a significant deleterious role in HIV progression. In agreement with our findings, a recent study reported several ribosomal formation genes including RPL27, PRS7, RPL24, RPS13 and RPL10L was down regulated in NK cells from HIV infected individuals [56]. Our most interesting findings were the identification of gene networks which were highly associated with nonprogression status in VNPs. These patients who retained a functional immune control of circulating viruses represented a very perplexing question pertinent to the LTNP status. Our findings hopefully provide a clue to resolve the mystery.

Nevertheless, we can not rule out the possibility that the changed transcription level of some gene found herein may reflect a consequence rather than a cause of low viral replication in LTNPs. The role of these candidate genes reported herein in controlling HIV-1 infection awaits further investigation.

## Conclusions

In summary, our integrative analysis of microarray data relevant to ECs and VNPs provides an overview of the biomarkers and transcriptional patterns in nonpathogen individuals compared with chronic progressors. We observed that HIV controllers had reduced immune activation and interferon response, which collaborates the concept that interferons might play a deleterious role in AIDS disease progression. Moreover, we have identified several key genes responsible for the non-progressive status, some of which were validated in cell lines. We also detected several pathways that may be related to the exacerbation of immunosuppression in AIDS patients, i.e., blockade of translation and dysfunction of T cell homeostasis. Altogether, our integrative genome-wide analysis has provided new knowledge for HIV-1 pathogenesis and immune intervention of the disease progression of AIDS.

## Additional files

**Additional file 1.** Clinical parameters of patients involved.

**Additional file 2.** DEGs in NPs compared with PPs in MADNT.

**Additional file 3.** DEGs in NPs compared with PPs in CD8+ T cells.

**Additional file 4.** Pathways enriched in CD8+ T cells from ECs analyzed by GSEA.

**Additional file 5.** Genes in modules correlated with protection in CD8+ T cells (EC).

## Abbreviations

HIV-1: human immunodeficiency virus
AIDS: acquired immunodeficiency syndrome
GEO: Gene Expression Ominbus
LTNP: long-term nonprogressors
EC: elite controllers
DEG: differentially expressed genes
cART: combination anti-retroviral therapy
VC: viremic controllers
VNP: viremic nonprogressors
NP: normal progressors
SNP: single nucleotide polymorphism
IL-2: interleukin 2
RMA: Robust Multi-Array Analysis
ISGs: interferon-stimulated genes
FDR: false discovery rate
GO: gene ontology
KEGG: Kyoto Encyclopedia of Genes and Genomes
DAVID: Database for Annotation, Visualization and Integrated Discovery
MADNT: meta-analysis derived nonprogressor transcriptome
GSEA: gene set enrichment analysis
WGCNA: weighted gene coexpression network analysis
PPI: protein–protein networks
PBMC: peripheral blood mononuclear cell
IRF7: IFN regulatory factor 7
IQR: inner-quartile range
DMEM: Dulbecco’s modified Eagle medium
